# Posture‐Related Regional Homogeneity Abnormalities in Cerebral Small Vessel Disease: A Multi‐Position MRI Study

**DOI:** 10.1002/cns.71140

**Published:** 2026-09-07

**Authors:** Yukun Zhang, Qi Dai, Weinv Fan, Hu Yu, Liang Hu, Shenyu Fan, Ling Ni, Danying Ma, Disen Wang, Wesley Surento, Xin Zhang, Rongfeng Qi, Jianjun Zheng, Bing Zhang

**Affiliations:** ^1^ Department of Radiology, Nanjing Drum Tower Hospital, Affiliated Hospital of Medical School Nanjing University Nanjing China; ^2^ Department of Psychology Nanjing University Nanjing China; ^3^ Department of Radiology Ningbo No. 2 Hospital Ningbo China; ^4^ Zhejiang Engineering Research Center for New Technologies and Applications of Helium‐Free Magnetic Resonance Imaging Ningbo China; ^5^ Department of Neurology Ningbo No. 2 Hospital Ningbo China; ^6^ Department of Biomedical Informatics and Medical Education University of Washington Seattle Washington USA; ^7^ Jiangsu Key Laboratory for Cardiovascular Information and Health Engineering Medicine Nanjing China; ^8^ Nanjing Key Laboratory for Cardiovascular Information and Health Engineering Medicine Nanjing China

**Keywords:** body position, cerebral small vessel disease, functional magnetic resonance imaging, regional homogeneity, spontaneous brain activity

## Abstract

**Introduction:**

Cerebral small vessel disease (CSVD) is associated with altered spontaneous brain activity on resting‐state functional magnetic resonance imaging (rs‐fMRI). However, MRI studies usually overlook the potential effects of postural change. This study characterized posture‐related alterations in spontaneous brain activity in CSVD patients.

**Materials and Methods:**

Forty‐one CSVD patients and 30 normal controls (NCs) underwent multi‐position MRI, including upright and supine rs‐fMRI. Amplitude of low‐frequency fluctuations (ALFF), fractional ALFF (fALFF), and regional homogeneity (ReHo) were calculated, and supine‐upright differences (Δ) were derived. Mixed‐design ANOVA assessed group, position, and interaction effects. Within‐group and between‐group differences were examined, followed by cognition‐related analyses.

**Results:**

The main effect of posture on ReHo was observed in frontal, temporal, and parietal cortices and deep gray nuclei. In the upright position, CSVD patients had higher ReHo in the right anterior cingulate gyrus than NCs, which was associated with worse global cognitive function and a higher composite CSVD score. CSVD patients also exhibited widespread posture‐related ReHo alterations. Regression analyses demonstrated that ΔReHo in the left hippocampus was positively associated with memory function, whereas ΔReHo in the right putamen and left calcarine cortex was associated with the composite CSVD score.

**Conclusion:**

Upright imaging and upright‐supine comparison may help unmask latent posture‐related ReHo abnormalities in patients with CSVD.

## Graphical abstract

Patients with CSVD showed higher upright ReHo in anterior cingulate gyrus (ACG) than NCs, and supine‐upright comparison revealed widespread ReHo alterations. Upright ACG ReHo and posture‐related ReHo changes in the hippocampus, putamen, and calcarine cortex were associated with poorer cognition and greater CSVD burden, suggesting that upright imaging may reveal latent posture‐related functional brain abnormalities.

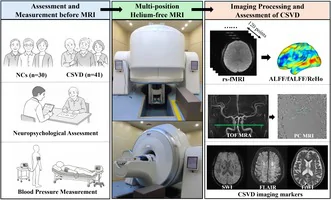

## 1 Introduction

Cerebral small vessel disease (CSVD) refers to pathological alterations affecting penetrating arterioles, capillaries, and small venules, resulting in widespread gray and white matter injury [1]. On magnetic resonance imaging (MRI), CSVD manifests as a spectrum of imaging markers, including white matter hyperintensities (WMHs), recent subcortical small infarcts (RSSIs), lacunes, enlarged perivascular spaces (EPVS), and cerebral microbleeds (CMBs) [2]. CSVD is a leading cause of lacunar stroke and a major pathological substrate of vascular cognitive impairment and dementia, thereby imposing an increasing burden in aging societies [3, 4]. As an important framework for elucidating the relationship between CSVD and cognitive dysfunction, functional MRI (fMRI) research has advanced the network disintegration model of vascular cognitive impairment [5, 6, 7, 8]. However, body posture—a physiological factor known to exert substantial modulation on brain functional organization—has largely been overlooked in CSVD research [9, 10, 11]. Accordingly, brain functional abnormalities identified in CSVD during supine fMRI alone may not fully capture posture‐sensitive neural regulation under more ecologically relevant conditions.

Upright posture requires multisensory integration and cortical control [12]. Although maintaining an upright stance is largely involuntary, aging increases the extent of cortical involvement needed even during stable standing [13]. Previous studies have shown that compared with young adults, older adults demonstrate higher electroencephalography (EEG) delta rhythms over the anterior cortical regions during an upright posture [13, 14]. In older adults, this upright posture is increasingly dependent on higher‐order cognitive processes such as executive function [15]. Further evidence indicates that WMH volume in older adults is positively correlated with higher EEG delta rhythm during upright posture [16]. However, most prior studies focused on healthy aging. It remains unclear whether posture‐dependent brain function is further impaired in patients with CSVD in the upright position or during supine‐upright transition.

Postural transitions induce systemic physiological adjustments involving blood pressure, cardiac output, blood flow redistribution, respiration, and autonomic activity [17, 18, 19, 20]. Cardiovascular regulation is increasingly recognized as an important contributor to posture‐related changes in brain functional organization [21, 22]. Resting‐state fMRI (rs‐fMRI) enables noninvasive assessment of brain function. Spontaneous brain activity metrics such as amplitude of low‐frequency fluctuations (ALFF), fractional ALFF (fALFF), and regional homogeneity (ReHo) have shown sensitivity to aging and WMH burden [23]. In CSVD patients, spontaneous brain activity abnormalities exhibit disrupted brain‐behavior coupling [24, 25]. Notably, dynamic cerebral autoregulation and neurovascular coupling have been reported to be impaired in CSVD patients [26], whereas patients with ischemic stroke exhibit reduced dynamic flow regulation during postural transitions [27, 28]. Therefore, we hypothesized that patients with CSVD would exhibit posture‐related alterations in spontaneous brain activity, which may be associated with impaired cardiovascular regulation. Given the limited prior evidence, the specific affected regions were identified using an exploratory whole‐brain analysis.

Recent advances in multi‐position helium‐free 1.5 T MRI systems, enabled by 180° magnet rotation, have made posture‐dependent neuroimaging feasible [29, 30]. Using this platform, we acquired upright and supine rs‐fMRI together with arterial carotid phase‐contrast MRI (PC MRI) in normal controls (NCs) and patients with CSVD to characterize spontaneous brain activity and static cerebral hemodynamic measures [31]. This study aimed to characterize posture‐related alterations in spontaneous brain activity in CSVD and their associations with cognition, CSVD burden, and cardiovascular regulation, providing further insight into the pathophysiological mechanisms underlying CSVD.

## 2 Materials and Methods

### 2.1 Participants

From September 2024 to September 2025, 48 patients with sporadic CSVD were prospectively recruited from Ningbo No. 2 Hospital. During the same period, 32 community‐dwelling normal controls (NCs) were also enrolled. The inclusion criteria for the CSVD group were as follows: (1) age > 50 years; (2) clinical presentation of post‐stroke lacunar syndrome, accompanied by subcortical small infarcts and/or WMHs (Fazekas score ≥ 2) [32]; and (3) MRI data available. The exclusion criteria for the CSVD group were: (1) stenosis > 50% in the major head or neck arteries; (2) severe brain disorders, including intracerebral hemorrhage, large territorial infarction, cerebrovascular malformation, intracranial aneurysm, moyamoya disease, brain tumor, or intracranial infection; (3) severe medical emergencies, such as heart, liver, lung, or renal failure; and (4) a history of cranial surgery. The inclusion criteria for the NC group were: (1) age > 50 years; (2) no presence of CSVD‐related imaging markers; (3) no contraindications to MRI. The exclusion criteria for the NC group were the same as those for the CSVD group.

From the initially recruited CSVD group, 5 patients were excluded due to arterial stenosis of > 50%, and 2 were excluded because of motion artifacts, leaving 41 patients for the final analysis. From the initially recruited NC group, 2 participants were excluded because of motion artifacts, leaving 30 NCs for the final analysis. For all participants, hypertension, hyperlipidemia, diabetes mellitus, smoking status, and alcohol consumption were recorded. Body mass index (BMI) was also calculated.

### 2.2 Image Acquisition Protocol

All participants underwent MRI using a multi‐position helium‐free 1.5 T MRI system (SPIN 1.5 T MRI, Xingaoyi Medical Equipment Co. Ltd., China) with a combined 16‐channel head‐and‐neck coil. Because upright scanning requires greater participant cooperation and postural stability, all participants underwent upright scanning first, followed by supine scanning [18]. The interval between the two acquisitions was approximately 5 min and was used for repositioning the MRI table. During upright scanning, the MRI magnet was tilted to 75° relative to the ground, representing a semi‐upright position. Hereafter, “upright” refers to this 75° scanning condition. Two 15 cm‐wide straps were used to secure the abdomen and legs of each participant to maintain stability on the scanning table. The MRI protocol included rs‐fMRI and PC MRI, both of which were acquired in upright and supine positions. In each position, rs‐fMRI was acquired first, followed by PC MRI. The remaining conventional sequences were acquired only in the supine position. Detailed scanning parameters are provided in Supporting Information.

### 2.3 Neuropsychological Assessment

Neuropsychological assessment was completed before the MRI examination. Global cognitive function was assessed using the Montreal Cognitive Assessment (MoCA) and the Mini‐Mental State Examination (MMSE). Information processing speed was assessed using the Trail‐Making Test Part A (TMT‐A). Executive function was assessed using the Trail‐Making Test Part B (TMT‐B) and the Digit Symbol Substitution Test (DSST). Memory function was assessed using the Auditory Verbal Learning Test (AVLT). Immediate recall (AVLT 1 + 2 + 3), short‐delay free recall (AVLT 4), and long‐delay free recall (AVLT 5) were used as three measures of memory. Language function was assessed using the Verbal Fluency Test (VFT) [32, 33]. Higher scores indicate better cognitive performance for all scales except TMT‐A and TMT‐B, for which higher scores indicate poorer performance. All test scores were converted to standardized *Z* scores separately within the NC and CSVD groups for subsequent statistical analysis.

### 2.4 Measurement of Hemodynamic Parameters

Peripheral hemodynamic parameters, including systolic blood pressure (SBP), diastolic blood pressure (DBP), mean arterial pressure (MAP), and heart rate (HR), were measured using a medical electronic sphygmomanometer. PC MRI‐derived cerebral hemodynamic parameters [31], including total arterial blood flow, arterial mean velocity, arterial maximum velocity, and arterial minimum velocity, were obtained as detailed in the Supporting Information. Supine‐upright differences (Δ) were calculated as supine minus upright values.

### 2.5 Image Preprocessing and Analysis of Spontaneous Brain Activity

Rs‐fMRI data were preprocessed using the Data Processing Assistant for Resting‐State fMRI Advanced Edition (DPARSFA) toolbox (version 6.1) [34] in MATLAB (The MathWorks Inc., Natick, MA, USA), which was also used to calculate ALFF, fALFF, and ReHo [35], as detailed in the Supporting Information.

Statistical analysis was conducted using Statistical Parametric Mapping Software (SPM12; http://www.fil.ion.ucl.ac.uk/spm) in MATLAB. To examine the effects of group, position, and their interaction on brain functional activity, a two‐way mixed‐design analysis of variance (ANOVA) was performed in SPM12 using a flexible factorial model. Group was modeled as a between‐subject factor, position as a within‐subject factor, and subject as a random factor to account for repeated measures. In addition, within‐group comparisons between the upright and supine positions were performed using paired *t*‐tests. Between‐group comparisons were performed using two‐sample *t*‐tests. Within‐group postural comparisons were adjusted for mean framewise displacement (FD), whereas between‐group comparisons were adjusted for age, sex, years of education, and mean FD. Multiple‐comparison correction was performed using the false discovery rate (FDR) method at the voxel level. Brain regions with a voxel‐wise FDR corrected *p* < 0.05 and a cluster size of at least 20 voxels were considered significant. The significant clusters were used as masks to extract corresponding spontaneous brain activity values and to calculate Δ.

### 2.6 Assessment of the CSVD Imaging Markers

CSVD‐related MRI markers, including number of lacunes, number of CMBs, WMH score, and EPVS score, were assessed according to established criteria by an associate chief radiologist with 10 years of experience. Details are provided in the Supporting Information. The composite CSVD score was also calculated [36, 37]. The scoring method was as follows: 1 point was assigned for the presence of CMBs, 1 point for the presence of lacunes or RSSIs, 1 point for Fazekas grade 2–3 WMHs, and 1 point for moderate‐to‐severe EPVS (number ≥ 11). The total score ranged from 0 to 4.

### 2.7 Statistical Analysis

All statistical analyses were performed using SPSS 21.0. Continuous variables were first tested for normality using the Kolmogorov–Smirnov test. Normally distributed data are presented as mean ± standard deviation (SD), and non‐normally distributed data are presented as median (interquartile range, IQR). Categorical variables are presented as frequency (percentage). Between‐group and within‐group postural comparisons were performed using independent‐sample *t*‐tests, Mann–Whitney *U* tests, paired‐sample *t*‐tests, Wilcoxon signed‐rank tests, or *χ* ^2^ tests, as appropriate.

Partial Spearman correlation analyses examined the associations between spontaneous brain activity and CSVD‐related imaging markers, adjusting for age, sex, years of education, and mean FD. Multiple linear regression (MLR) models examined associations between spontaneous brain activity and neuropsychological performance. Additional MLR models assessed associations between posture‐related Δspontaneous brain activity and Δhemodynamic measures. Between‐group differences in the associations between Δbrain activity and Δhemodynamic measures were tested by including group, the Δhemodynamic measures, and the group × Δhemodynamic measure interaction term in the models. All MLR models were adjusted for age, sex, years of education, mean FD, and composite CSVD score. Missing neuropsychological data were handled by pairwise deletion. Outliers were assessed for the variables included in each analysis; participants with values exceeding 3 standard deviations from the mean were excluded only from the corresponding analysis. For correlation analyses, FDR correction was applied across the regions showing significant differences in spontaneous brain activity for each corresponding analysis. Statistical significance was set at FDR‐corrected *p* < 0.05.

## 3 Results

### 3.1 Participant Characteristics

A total of 30 NCs were included in the study, including 12 males and 18 females, and the mean age was 60.30 ± 6.85 years. 41 patients with CSVD were enrolled, including 18 males and 23 females, with a mean age of 62.15 ± 7.00 years (Table 1). The proportion of smokers and the prevalence of hypertension and hyperlipidemia were significantly lower in NCs than in patients with CSVD. NCs showed better cognitive performance than CSVD patients (*p* < 0.05, Table 1).

**TABLE 1:** Demographic and clinical characteristics of NCs and CSVD patients.

	NCs (*n* = 30)	CSVD (*n* = 41)	Statistic value	*p*
Demographic				
Male, *n* (%)	12 (40.0%)	18 (43.9%)	0.327	0.744^c^
Age (years)	60.30 ± 6.85	62.15 ± 7.00	−1.092	0.279^a^
Education (years)	9.87 ± 4.30	8.32 ± 4.21	1.497	0.139^a^
Risk factors				
BMI	23.13 ± 2.67	23.58 ± 3.29	−0.767	0.446^a^
Smoking, *n* (%)	1 (3.3%)	10 (24.4%)	2.405	**0.016** ^c^
Drinking, *n* (%)	2 (6.7%)	9 (22.0%)	1.746	0.081^c^
Hypertension, *n* (%)	6 (20.0%)	25 (61.0%)	3.414	**0.001** ^c^
Diabetes, *n* (%)	3 (10.0%)	6 (14.6%)	0.576	0.565^c^
Hyperlipidemia, *n* (%)	5 (16.7%)	21 (51.2%)	2.964	**0.003** ^c^
Neuropsychological assessment				
MMSE	28.00 (26.00, 29.00)	27.00 (24.75, 28.00)	2.477	**0.013** ^b^
MoCA	25.50 (23.00, 27.00)	22.00 (18.00, 25.00)	2.959	**0.003** ^b^
AVLT1 + 2 + 3	19.97 ± 5.79	14.69 ± 5.41	3.759	**< 0.001** ^a^
AVLT4	7.47 ± 2.22	5.17 ± 2.89	3.515	**0.001** ^a^
AVLT5	7.77 ± 2.68	5.06 ± 3.18	3.645	**0.001** ^a^
VFT	14.00 (11.50, 19.50)	11.00 (9.00, 14.00)	2.795	**0.005** ^b^
TMT‐A	61.50 (44.50, 85.00)	89.00 (58.00, 130.00)	−2.975	**0.003** ^b^
TMT‐B	139.50 (106.25, 180.00)	216.00 (172.00, 265.00)	−3.935	**< 0.001** ^b^
DSST	35.30 ± 15.84	24.41 ± 13.30	2.941	**0.005** ^a^
CSVD‐related imaging markers				
Composite CSVD score	—	2 (1, 3)	—	—
Number of lacunes	—	1 (0, 2)	—	—
WMH score	—	2 (2, 2)	—	—
EPVS score	—	2 (1, 2)	—	—
Number of CMBs	—	0 (0, 2)	—	—
Head motion parameters of rs‐fMRI				
Upright mean FD	0.16 (0.14, 0.19)	0.18 (0.16, 0.20)	−1.404	0.165^b^
Supine mean FD	0.12 (0.11, 0.14)	0.14 (0.12, 0.15)	−1.529	0.131^b^
Δmean FD	−0.05 (−0.05, −0.03)	−0.04 (−0.06, −0.02)	−0.174	0.862^b^

*Note:* Δ was defined as supine minus upright. Bold values indicate statistical significance (*p* < 0.05).

Abbreviations: AVLT 1 + 2 + 3, Auditory Verbal Learning Test immediate recall; AVLT 4, AVLT short‐delay free recall; AVLT 5, AVLT long‐delay free recall; BMI, body mass index; CMBs, cerebral microbleeds; CSVD, cerebral small vessel disease; DSST, Digit Symbol Substitution Test; EPVS, enlarged perivascular space; FD, framewise displacement; MMSE, Mini‐Mental State Examination; MoCA, Montreal Cognitive Assessment; NCs, normal controls; TMT‐A, Trail‐Making Test Part A; TMT‐B, Trail‐Making Test Part B; VFT, Verbal Fluency Test; WMH, white matter hyperintensity.

^a^ Independent‐sample *t*‐test.

^b^ Mann–Whitney *U* test.

^c^ *χ* ^2^ Test.

### 3.2 Main Effects and Interaction Effects of Group and Position

**FIGURE 1:**
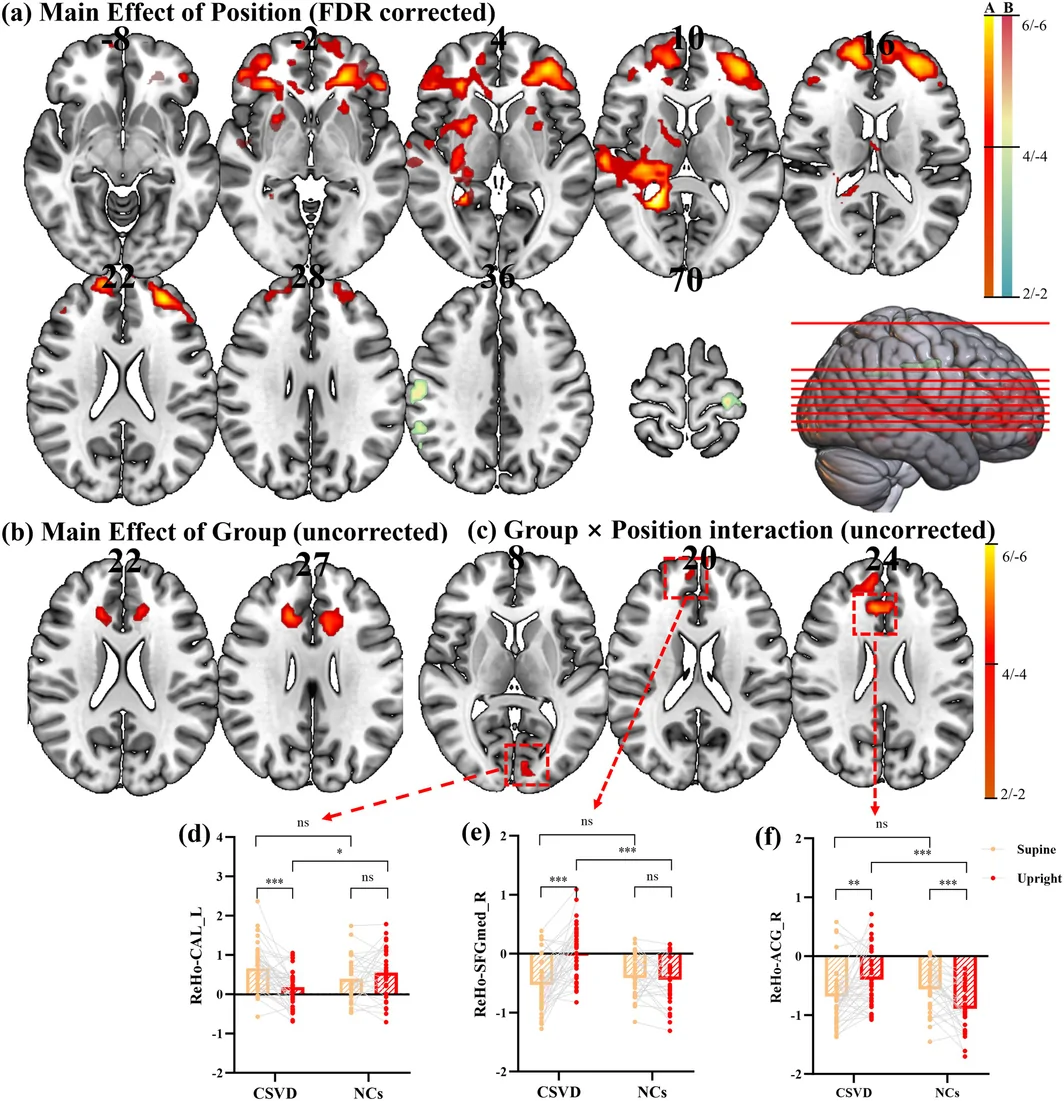
Main Effects and Interaction Effects of Group and Position. (a) The main effect of position (*p* < 0.05, FDR corrected). Color bar A shows increased ReHo, whereas color bar B shows decreased ReHo in the upright position relative to the supine position. The values on the color bars represent the corresponding statistical values. (b) The exploratory main effect of the group (*p* < 0.001, uncorrected). (c) The exploratory group × position interaction analysis (*p* < 0.001, uncorrected). (d–f) Comparison of the mean ReHo values extracted from the left CAL, right SFGmed, and right ACG. ACG, anterior cingulate gyrus; CAL, calcarine; CSVD, cerebral small vessel disease; NCs, normal controls; ReHo, regional homogeneity. SFGmed, the medial part of the superior frontal gyrus. Significance symbols: *0.01 < *p* < 0.05; **0.001 < *p* < 0.01, ***p < 0.001; ns, not significant.

After covariate adjustment, no spontaneous brain activity metric showed a significant main effect of group after FDR correction. The main effect analysis of position showed that, after covariate adjustment, ReHo was significantly altered in 10 brain regions (*p* < 0.05, FDR corrected; Figure 1 and Table S1). Further analysis of the group × position interaction showed that, after covariate adjustment, no spontaneous brain activity metric retained significance after FDR correction (*p* > 0.05, FDR corrected).

Additional exploratory analyses of the three spontaneous brain activity metrics were performed at an uncorrected voxel‐wise threshold of *p* < 0.001. These analyses suggested a possible main effect of group in the bilateral anterior cingulate gyrus (ACG), and possible group × position interaction effects in the left calcarine cortex (CAL), right ACG, and right medial superior frontal gyrus (SFGmed) for ReHo (*p* < 0.001; Figure 1b,c and Table S2). Post hoc analyses of mean ReHo values within the three exploratory interaction clusters showed that, in the left CAL, upright ReHo was lower than supine ReHo in the CSVD group, and upright ReHo was also lower in CSVD than in NCs. In the right SFGmed, upright ReHo was higher than supine ReHo in the CSVD group, and upright ReHo was also higher in CSVD than in NCs. In the right ACG, upright ReHo increased in the CSVD group but decreased in the NC group relative to the supine position, and upright ReHo was higher in CSVD than in NCs (all *p* < 0.05; Figure 1d–f).

### 3.3 Between‐Group Comparisons in the Upright and Supine Positions and Their Clinical Relevance

**FIGURE 2:**
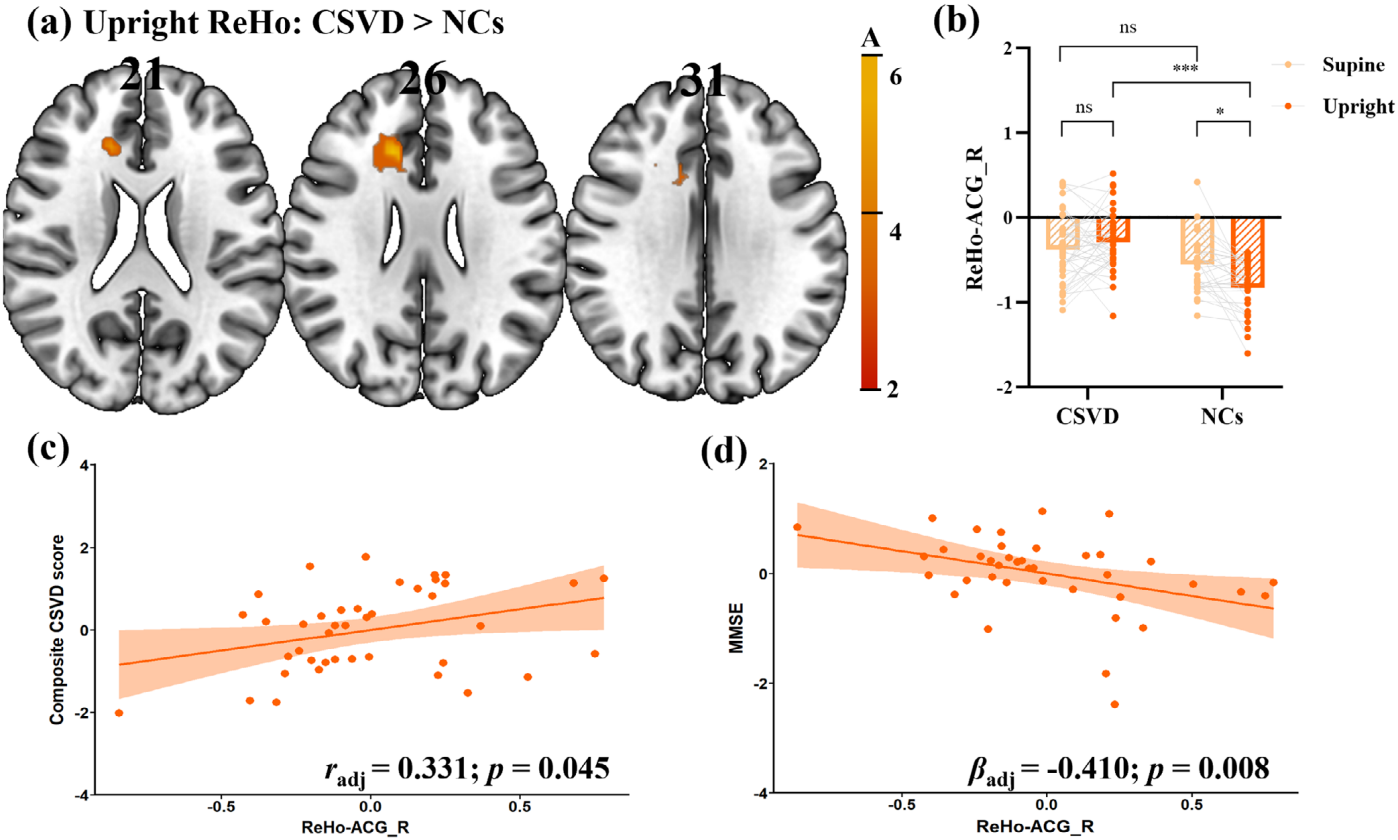
Comparison of spontaneous brain activity between the CSVD and NC groups in the upright position and association analyses. (a) Between‐group comparison of ReHo in the CSVD and NC groups under upright conditions (*p* < 0.05, FDR corrected). Color bar A indicate increased ReHo in CSVD, and the values on the color bars represent the corresponding statistical values. (b) Comparison of the mean ReHo values extracted from the right ACG. (c, d) Associations of ReHo values in the right ACG with composite CSVD score and MMSE score. Both axes in the scatter plots show residualized values after adjustment for covariates. ACG, anterior cingulate gyrus; CSVD, cerebral small vessel disease; MMSE, Mini‐Mental State Examination; NCs, normal controls; ReHo, regional homogeneity. Significance symbols: *0.01 < *p* < 0.05; **0.001 < *p* < 0.01, ***p < 0.001; ns, not significant.

In the supine position, no significant between‐group differences were found among the three spontaneous brain activity metrics between the CSVD and NC groups after covariate adjustment (*p* > 0.05, FDR corrected). In the upright position, after covariate adjustment, only ReHo in the right ACG was significantly higher in the CSVD group than in the NC group (*p* < 0.05, FDR corrected; Figure 2a and Table S3). In the right ACG, upright ReHo was lower than supine ReHo in NCs, whereas upright ReHo was higher in patients with CSVD than in NCs (*p* < 0.05; Figure 2b). Partial correlation analysis showed that ReHo in the right ACG in the upright position was positively associated with the composite CSVD score (*r*_adj_ = 0.331, *p* = 0.045; Figure 2c). One participant with CSVD was excluded as an outlier because of an extremely low MMSE score. After covariate adjustment, MLR analysis showed that ReHo in the right ACG was negatively associated with MMSE score (*β*_adj_ = −0.410, *p* = 0.008; Figure 2d). Because only one significant cluster was identified, no additional correction was applied for the correlation analysis. Additional MLR model statistical results are provided in Table S4.

### 3.4 Supine‐Upright Comparisons in NCs and CSVD and Their Clinical Relevance

**FIGURE 3:**
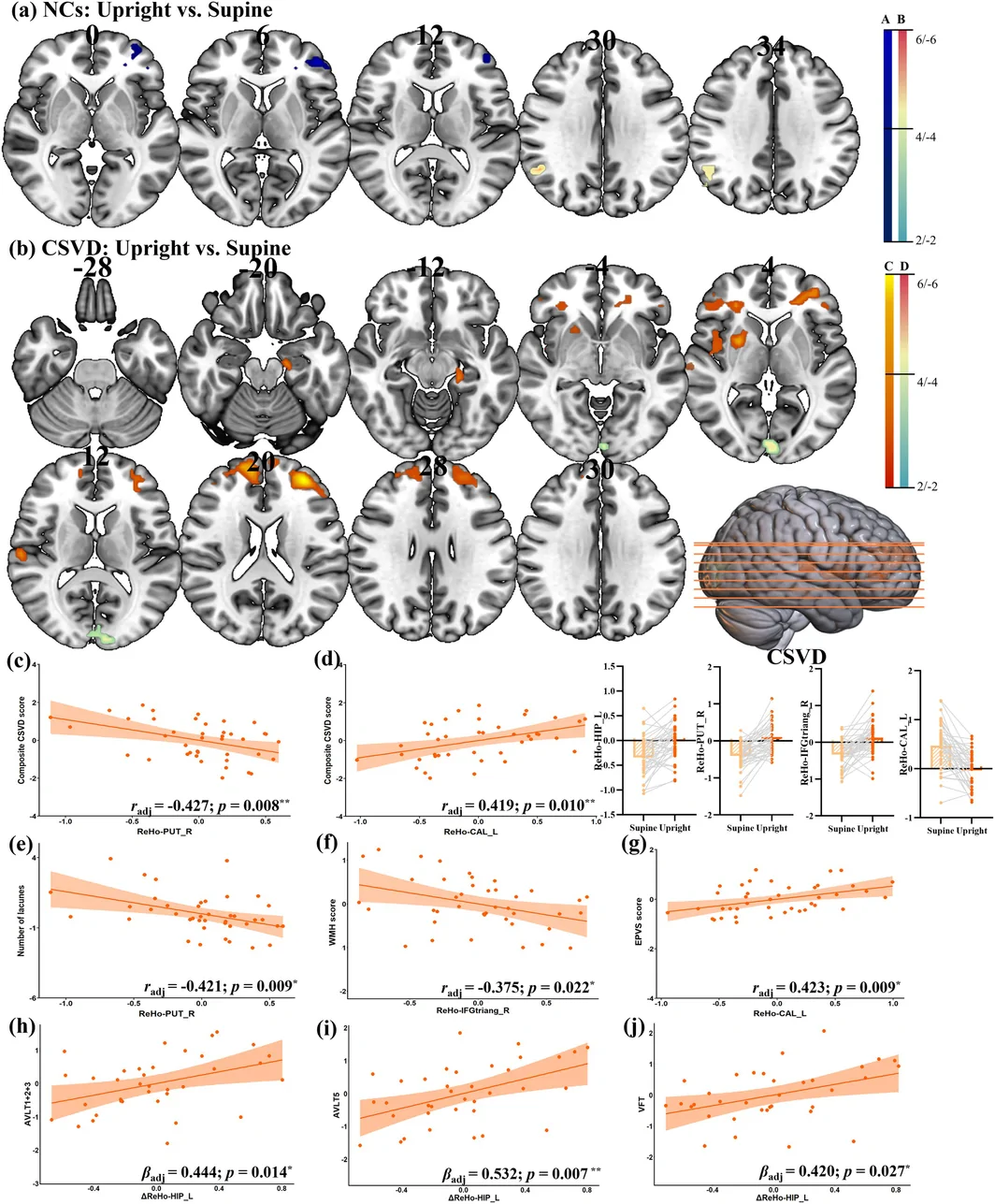
Posture‐related ReHo alterations and their clinical relevance. (a, b) Significant ReHo alterations between the upright and supine positions in NCs and CSVD after FDR correction. Color bar A and C show increased ReHo, whereas color bar B and D show decreased ReHo in the upright relative to the supine position. The values on the color bars represent the corresponding statistical values. (c, d) Associations of ΔReHo in the right PUT and left CAL with the composite CSVD score. (e–g) Associations of ΔReHo in the right PUT, right IFGtriang and left CAL with the CSVD imaging markers. (h–j) Associations of ΔReHo in the left HIP with cognitive performance. Δ was defined as supine minus upright. Both axes in the scatter plots show residualized values after adjustment for covariates. AVLT 1 + 2 + 3, Auditory Verbal Learning Test immediate recall; AVLT 5, AVLT long‐delay free recall; CAL, calcarine cortex; CSVD, cerebral small vessel disease; EPVS, enlarged perivascular spaces; HIP, hippocampus; IFGtriang, triangular part of the inferior frontal gyrus; NCs, normal controls; PUT, putamen; ReHo, regional homogeneity; VFT, Verbal Fluency Test; WMHs, white matter hyperintensities. Significance symbols: *p < 0.05, uncorrected; **p < 0.05, FDR‐corrected.

In the NC group, compared with the supine position, the upright position showed increased ReHo in the left triangular part of the inferior frontal gyrus (IFGtriang) and decreased ReHo in the right angular gyrus (ANG) (*p* < 0.05, FDR corrected; Figure 3a and Table S3). In the CSVD group, a total of 8 clusters survived FDR correction, seven of which showed higher ReHo in the upright than in the supine position. These clusters included the left hippocampus (HIP), right insula (INS), right putamen (PUT), bilateral middle frontal gyri (MFG), bilateral IFGtriang, bilateral superior frontal gyri (SFG), and right superior temporal gyrus (STG). Only the left CAL showed lower ReHo in the upright compared with the supine position (*p* < 0.05, FDR corrected; Figure 3b and Table S3). No significant upright‐supine differences in ALFF or fALFF were observed in either the NC or CSVD group (*p* > 0.05).

ReHo values were extracted from 2 brain clusters in NCs and 8 brain clusters in CSVD under both positions, and supine‐upright differences (Δ) were then calculated. After covariate adjustment, MLR analyses showed that no significant association between ΔReHo and cognitive test scores was found in NCs. In the CSVD group, partial correlation analyses revealed that ΔReHo in the right PUT (*r*_adj_ = −0.427, *p* = 0.008) and in the left CAL (*r*_adj_ = 0.419, *p* = 0.010) were significantly associated with the composite CSVD score; both associations survived FDR correction (Figure 3c,d). ΔReHo in the right PUT was negatively correlated with the number of lacunes (*r*_adj_ = −0.421, *p* = 0.009), ΔReHo in the right IFGtriang was negatively associated with WMH score (*r*_adj_ = −0.375, *p* = 0.022), and ΔReHo in the left CAL was positively associated with EPVS score (*r*_adj_ = 0.423, *p* = 0.009; all uncorrected; Figure 3e–g). After covariate adjustment, MLR analyses showed that ΔReHo in left HIP was positively associated with AVLT1 + 2 + 3 (*β*_adj_ = 0.444, *p* = 0.014), AVLT5 (*β*_adj_ = 0.532, *p* = 0.007), and VFT (*β*_adj_ = 0.420, *p* = 0.027), with only the association with AVLT5 surviving FDR correction (Figure 3h–j). Additional MLR model statistics are provided in Table S4.

### 3.5 Associations of Posture‐Related Brain Functional Changes With Hemodynamic Measures

**FIGURE 4:**
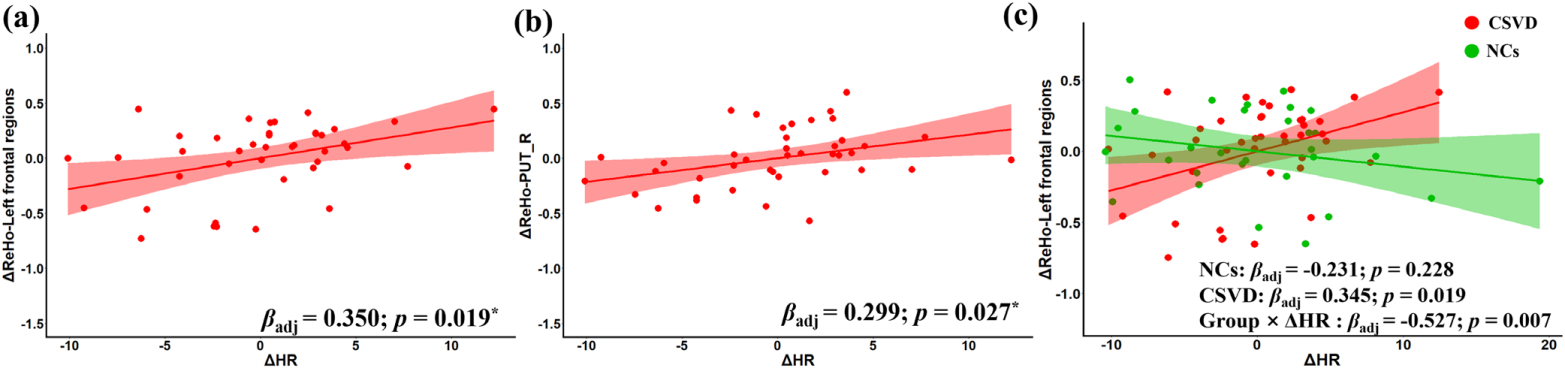
Associations of posture‐related ΔReHo with Δhemodynamic parameters. (a, b) Associations between cluster‐wise ΔReHo and Δhemodynamic parameters in the CSVD group. (c) Group × ΔHR interaction for ΔReHo in the left frontal regions. Δ was defined as supine minus upright. Both axes in the scatter plots show residualized values after adjustment for covariates. CSVD, cerebral small vessel disease; HR, heart rate; NCs, normal controls; PUT, putamen; ReHo, regional homogeneity. Significance symbols: *p < 0.05, uncorrected.

Using the clusters as binary masks, ReHo values were extracted from all 10 masks in the main effect of position (Figure 1a and Table S1), and supine‐upright differences (Δ) were calculated. In the NC group, MLR analysis showed that, after covariate adjustment, no significant associations were found between ΔReHo and the Δhemodynamic measures. In the CSVD group, four different participants were identified as outliers: one for ΔReHo in each of Clusters 5, 6, and 8, and one for Δtotal arterial flow. Each participant was excluded only from the corresponding analysis. After covariate adjustment, MLR analyses showed that ΔReHo in the left frontal regions (Cluster 3; *β*_adj_ = 0.350, *p* = 0.019) and right PUT (Cluster 4; *β*_adj_ = 0.299, *p* = 0.027) were positively associated with ΔHR at an uncorrected threshold (Figure 4a,b). Additional MLR model statistics are provided in Table S4. Further MLR with a group × ΔHR interaction analysis showed that, after adjustment covariates, the association between ΔReHo in the left frontal regions and ΔHR differed significantly between groups (*β*_adj_ = −0.527, *p* = 0.007; Figure 4c and Table S5), being significant in the CSVD group but not in the NC group.

In addition, the ΔMAP was significantly lower in the CSVD group than in the NC group (*p* = 0.042), while other cerebral and peripheral hemodynamic measures did not differ significantly between the two groups (*p* > 0.05; Table S6).

## 4 Discussion

To our knowledge, this is the first multi‐position MRI study to investigate posture‐related alterations in spontaneous brain activity in CSVD patients. Across all participants, posture broadly affected ReHo in cortical and deep gray matter regions. Moreover, in the upright position, CSVD patients showed higher ReHo in the right ACG than NCs, which was associated with lower MMSE scores and higher composite CSVD scores. In CSVD patients, posture‐related ΔReHo in the left HIP was positively associated with AVLT5 scores, whereas ΔReHo in the right PUT and left CAL cortex were significantly associated with the composite CSVD score. Notably, the association between ΔReHo in the left frontal regions and ΔHR differed significantly between groups, being significant in the CSVD group but not in the NC group.

The upright posture depends on the integration of vestibular, visual, and proprioceptive inputs [12]. This study found that, in the upright posture, ReHo in the right ACG was significantly higher in the CSVD group than in the NC group. Previous studies have shown that structural and functional abnormalities may occur in the ACG in patients with CSVD [38, 39]. The right ACG also showed a possible group × posture interaction in the exploratory analysis. ReHo in the right ACG decreased from the supine to upright position in the NC group, whereas it increased in the CSVD group. In postural control, the ACG is assumed to play a functional role in sensory information integration and error detection [40, 41]. In NCs, lower ACG ReHo in the upright position may reflect more efficient postural regulation. Correlation analyses revealed that increased ReHo in the right ACG was associated with poorer global cognition and a higher CSVD burden. Taking into account previous evidence of ACG abnormalities in CSVD patients, this finding may indicate altered or less efficient involvement of the ACG during the upright position [23, 42].

Previous EEG and magnetoencephalography (MEG) studies have shown that, in healthy adults, standing can rapidly increase cortical high‐frequency activity relative to the supine position [43, 44]. In all participants, the main effect of posture on ReHo showed that upright‐related increases were mainly distributed in the frontal, temporal, occipital, and deep gray matter nuclei, and decreases primarily in parietal regions. These findings indicate that postural change broadly modulates local spontaneous brain activity in older adults. Specifically, NCs showed relatively limited posture‐related ReHo changes, with increased ReHo only in the left IFGtriang. Unlike NCs, patients with CSVD also showed more widespread posture‐related ReHo alterations. ReHo increased in the bilateral SFG, bilateral MFG, bilateral IFGtriang, right STG, right INS, right PUT, and left HIP, but decreased in the left CAL. Moreover, the right SFGmed showed a possible group‐by‐posture interaction. Previous studies by Nóbrega and George have shown that the prefrontal cortex in older adults is closely involved in sensorimotor regulation and can be activated even during relatively simple balance tasks [45, 46]. Hence, these findings suggest that NCs and CSVD patients may recruit higher‐order cognitive control to support postural maintenance in the upright position [47]. Notably, however, CSVD patients showed more extensive frontal involvement, including bilateral frontal regions. In this context, the broader and more bilateral frontal activation observed in CSVD may reflect increased neural recruitment, possibly compensatory, in response to abnormalities in the sensorimotor system [48, 49]. In addition, the present findings were mainly confined to ReHo, suggesting that postural change may be more closely associated with local temporal coordination of spontaneous neural activity than with alterations in the amplitude of low‐frequency fluctuations.

Further correlation analyses indicated that CSVD patients with poorer memory showed a greater increase in hippocampal ReHo in the upright than in the supine position, and patients with greater CSVD burden showed a more marked increase in putaminal ReHo and a greater decrease in calcarine ReHo in the upright than in the supine position. The PUT is related to automatic motor regulation during postural control [50]. The HIP mainly supports spatial orientation and body‐related spatial representation [51]. The CAL mainly participates in visual processing and visuospatial integration [52]. This finding suggests that patients with CSVD require region‐specific recruitment of posture‐related brain regions to maintain simple upright balance [16], but this recruitment pattern may partly reflect disease‐related dysfunction. Previous studies have also reported abnormalities in these regions in CSVD patients [35, 53]. Taken together, during the transition from supine to upright position, healthy individuals may regulate postural adaptation through adaptive increases or decreases in regional brain activity. In contrast, in patients with CSVD, some brain regions involved in postural regulation may show compensatory functional enhancement, whereas other abnormal regions may exhibit altered activity associated with cognitive decline or increased CSVD burden.

In the present study, we assessed posture‐related changes in static hemodynamic parameters as indirect markers of hemodynamic regulation. Except for ΔMAP, most cerebral and peripheral hemodynamic measures did not differ significantly between groups, suggesting limited overt between‐group differences in static hemodynamic responses. Further association analyses suggested that ΔReHo in the left frontal regions and right PUT may be related to hemodynamic alterations in the CSVD group. Interaction analysis showed that the association between left frontal ΔReHo and ΔHR was stronger in the CSVD group than in the NC group. These results indicate that despite similarities in the magnitude of static HR regulation between the two groups, a significant association between ΔHR and left frontal ΔReHo was observed only in CSVD patients. This could be explained by the redistribution of blood and the reduction in cerebral perfusion that occur during the transition from the supine to the upright position. Healthy individuals can maintain cerebral perfusion through cerebrovascular autoregulation and compensatory activation of the sympathetic nervous system, mainly by increasing HR and vascular resistance [54]. In contrast, due to impaired cerebrovascular autoregulation and autonomic nervous system dysfunction [55, 56, 57], patients with CSVD may still show a similar apparent magnitude of HR regulation as normal controls, but this regulation may require greater involvement of the prefrontal cortex [58, 59]. This may explain why frontal ReHo increased in the upright position and was significantly associated with ΔHR.

Overall, from a clinical translational perspective, multi‐position MRI remains an emerging specialized technique with limited accessibility and is unlikely to replace conventional supine MRI in the near term. However, its helium‐free design supports both routine supine and upright scanning, enabling additional posture‐dependent physiological and brain functional information without compromising standard MRI applications [29, 30]. In this study, no significant between‐group ReHo differences were observed in the supine position, whereas upright imaging revealed abnormal ReHo in the right ACG and broader posture‐related ReHo alterations in patients with CSVD, some of which were associated with cognition and CSVD burden. Larger multicenter studies are needed to validate reproducibility, clinical benefit, and cost‐effectiveness.

Several limitations of this study should be noted. First, the sample size was relatively small, potentially limiting statistical power and robustness of findings. Second, head motion is generally greater during upright than supine rs‐fMRI. Although head motion was strictly controlled during preprocessing and mean FD was included as a covariate, residual motion effects during upright scanning could not be completely excluded. Third, the cerebellum was not included in the analysis because it was not fully acquired in its entirety for some participants due to the limited slice coverage of rs‐fMRI. Fourth, to ensure participant comfort and reduce head motion, the angle between the MRI magnet and the ground was set at 75° during upright scanning, rather than a fully vertical 90°. Fifth, all MRI scans were acquired using fixed postural and sequence orders, with each sequence acquired only once. Therefore, potential order effects cannot be completely excluded, and measurement reproducibility could not be assessed. Finally, some selection bias may have been present in the CSVD group, because patients with more severe conditions were excluded due to the risk of falling during upright scanning. This may have affected the representativeness of the study sample.

In conclusion, supine‐upright postural change broadly affected ReHo in older participants. CSVD patients showed higher right ACG ReHo than NCs in the upright position, while postural transition induced widespread ReHo alterations within CSVD. Higher upright ACG ReHo and ΔReHo in the HIP, PUT, and CAL were associated with poorer cognition and greater CSVD burden. In addition, posture‐related hemodynamic changes may be associated with increased frontal ReHo in CSVD patients. These findings suggest that upright imaging and supine‐upright comparison assessment using multi‐position MRI may help reveal latent posture‐related functional brain abnormalities in CSVD patients.

## Author Contributions

**Yukun Zhang, Qi Dai:** conceptualization, methodology, data curation, formal analysis, writing – original draft. **Weinv Fan, Hu Yu:** data curation, software. **Liang Hu:** resources, supervision, visualization. **Shenyu Fan, Ling Ni:** investigation, validation. **Danying Ma, Disen Wang:** data curation, software. **Wesley Surento:** writing – review and editing. **Xin Zhang, Jianjun Zheng:** supervision, visualization. **Rongfeng Qi**, **Bing Zhang:** conceptualization, supervision, writing – review aand editing, funding acquisition.

## Funding

The study was supported by National Natural Science Foundation of China (No. 82271965, 82330059); Jiangsu Provincial Health Commission General Science Program (No. M2024057); the National Science and Technology Innovation 2030—Major program of “Brain Science and Brain‐Like Research” (No. 2022ZD0211800); 2025 Nanjing Outstanding Postdoctoral Research Project (SE7000202503); Talent Research Start‐up Fund of Nanjing Drum Tower Hospital (No. RC2024‐055); 2024 Open Research Fund Project of the Zhejiang Engineering Research Center (No. 2024GCLX09); and 2025 Key Medical Research Project of Jiangsu Provincial Health Commission (No. K2025054). The funding sources had no role in the writing of the report or the decision to submit the paper for publication.

## Ethics Statement

This study was approved by the Ethics Committee of Ningbo No. 2 Hospital (PJ‐NBEY‐KY‐2024‐130‐02) and conducted in accordance with the Declaration of Helsinki.

## Conflicts of Interest

The authors declare no conflicts of interest.

## Supporting information


**Table S1:** Main effect of position on ReHo.
**Table S2:** Main effect of group and group × position interaction on ReHo at an uncorrected threshold.
**Table S3:** Between‐group and within‐group postural differences in ReHo.
**Table S4:** Statistical summary of multiple linear regression models.
**Table S5:** Group × ΔHR Interaction on Left Frontal ΔReHo.
**Table S6:** Hemodynamic measures of NCs and CSVD.

## Data Availability

Original data generated in this study primarily consist of human MRI images and clinical data acquired at our hospital and are managed in accordance with the hospital's clinical research ethics policies. Therefore, the data cannot be made publicly available. However, they may be obtained from the corresponding author upon reasonable and formal request.
