# Supplementary material for: Posture‐Related Regional Homogeneity Abnormalities in Cerebral Small Vessel Disease: A Multi‐Position MRI Study
Source: CNS Neurosci Ther. 2026 Sep 7;32(9):e71140. doi: 10.1002/cns.71140 (PMC13551013; doi:10.1002/cns.71140)
Supplement: Supplementary file 1 [file CNS-32-e71140-s001.docx]

**Supporting Information Text**

**Supplementary Methods** **-** Detailed methods for MRI acquisition, measurement of spontaneous brain activity metrics, hemodynamic evaluations, and assessment of CSVD imaging markers.

**2.1 | Image acquisition protocol**

Resting-state functional MRI (rs-fMRI; slice thickness, 4 mm; field of view (FOV), 240 mm × 240 mm; matrix, 64 × 64; echo time (TE), 24 ms; repetition time (TR), 2500 ms; flip angle, 90°; number of slices, 24; 120 time points; and acquisition time, 302 s). Phase-contrast MRI (PC MRI; TR, 21 ms; TE, 11.5 ms; FOV, 160 mm × 160 mm; acquisition matrix, 256 × 128; flip angle, 20°; and velocity measurements at 25 time points during one cardiac cycle). T2-weighted imaging (T_2_WI; TR, 4090 ms; TE, 102 ms; FOV, 250 × 200 mm; acquisition time, 49 s), susceptibility weighted imaging (SWI; TR, 54 ms; TE, 40 ms; FOV, 230 × 184 mm; acquisition time, 166 s), time-of-flight MR angiography (TOF MRA; TR, 26 ms; TE, 7 ms; FOV, 250 × 175 mm; acquisition time, 297 s), T2-weighted fluid-attenuated inversion recovery (FLAIR; TR, 8000 ms; TE, 104 ms; FOV, 240 × 192 mm; acquisition time, 176 s), diffusion-weighted imaging (DWI; TR, 3245 ms; TE, 89 ms; FOV, 240 × 240 mm; acquisition time, 78 s), and three-dimensional T1-weighted imaging (3D T_1_WI; TR, 10 ms; TE, 3.4 ms; FOV, 230 × 230 mm; acquisition time, 255 s). rs-fMRI was acquired using a gradient-echo sequence. PC MRI slice was positioned perpendicular to the C4 segment of the bilateral internal carotid arteries and the basilar artery. The velocity encoding value was set to 120-150 cm/s.

**2.2 | Measurement of spontaneous brain activity metrics**

2.2.1 rs-fMRI data preprocessing

Resting-state fMRI data were preprocessed using the DPARSFA toolbox^[1]^ (version 6.1) in MATLAB (The MathWorks, Inc., Natick, MA, USA). The preprocessing steps were as follows. (1) The first 5 time points were discarded to reduce the effects of magnetic field instability and initial subject adaptation. A total of 115 time points were retained for further analysis. (2) Slice timing correction was performed. An interleaved acquisition order was used, and the first slice was set as the reference slice. (3) Head motion correction was then performed. Images at each time point were realigned to the reference image. Mean framewise displacement (FD) was recorded as a head motion parameter. Participants were excluded if their head motion exceeded 3 mm in translation or 3° in rotation. (4) Nuisance covariates were regressed out, including WM signals, cerebrospinal fluid (CSF) signals, and head motion parameters. (5) The mean BOLD image was co-registered to the T1WI to obtain the linear transformation parameters. The T1WI was then segmented, and the resulting deformation fields were used to normalize the fMRI to Montreal Neurological Institute (MNI) space. The voxel size was resampled to 3 × 3 × 3 mm³. Data with poor normalization quality were excluded after quality control. Both upright and supine rs-fMRI data were registered to the 3D T1WI acquired in the supine position. This approach was supported by a previous study reporting no significant regional brain volume differences between upright and supine positions in healthy individuals ^[2]^. (6) Spatial smoothing was applied to the normalized images to improve the signal-to-noise ratio. (7) Temporal band-pass filtering was performed with a frequency range of 0.01–0.08 Hz.

2.2.2 ALFF, fALFF, and ReHo analysis

ALFF, fALFF, and ReHo were calculated using the DPARSFA toolbox^[1]^, available on the MATLAB platform. ALFF and fALFF were calculated based on images after preprocessing steps (1)-(6). Band-pass filtering was not applied before ALFF/fALFF calculation. First, the mean square root of the power spectrum of the BOLD signal within the low-frequency range (0.01–0.08 Hz) was calculated for each voxel to obtain the ALFF value. Then, the ratio of power within the low-frequency range to that across the entire frequency range was calculated as the fALFF value. ReHo was calculated based on images after preprocessing steps (1)-(5) and (7). To avoid the influence of spatial smoothing on local synchronization, ReHo was calculated before smoothing. The ReHo value of each voxel was divided by the global mean ReHo value to generate a normalized ReHo map. The normalized ReHo map was then spatially smoothed with a 4-mm kernel. To reduce intersubject variability in overall signal amplitude and to facilitate between-subject comparison, ALFF, fALFF, and ReHo maps were further converted into standardized Z-score maps.

**2.3 | Measurement of hemodynamic parameters**

2.3.1 Peripheral hemodynamic measures

Blood pressure was measured at the right brachial artery using a medical electronic sphygmomanometer (YE900, Yuwell, China). Systolic blood pressure (SBP), diastolic blood pressure (DBP), and heart rate (HR) were recorded. Measurements were obtained in both the upright and supine positions. Before each measurement, participants rested quietly for 3–5 min to ensure stable and accurate results. MAP was calculated as $DBP+1/{3 (SBP-DBP)}$.

2.3.2 Cerebral hemodynamics measures

All PC MRI images were transferred to the Xingaoyi image post-processing workstation and analyzed using ROI flow-velocity curve software (FlowRoi 1.0.24, China). The analysis was performed by a senior imaging technologist with 5 years of experience in imaging equipment operation. On the PC MRI images, regions of interest (ROIs) were manually drawn along the vessel boundaries of the bilateral internal carotid arteries (ICAs) and the basilar artery (BA). The ROI area was required to cover at least 80% of the vascular cross-sectional area. During one cardiac cycle, the mean flow, mean velocity, maximum velocity, and minimum velocity within the ROI were calculated for the bilateral ICAs and the BA. The sum of the mean flow in the bilateral ICA and the BA was defined as total arterial blood flow ^[3]^. The mean values of mean velocity, maximum velocity, and minimum velocity across the three arteries were defined as arterial mean velocity, arterial maximum velocity, and arterial minimum velocity, respectively.

**2.4 | Assessment of CSVD imaging markers**

Lacunes were identified as small cavity-like lesions with a central hypointense signal and a surrounding hyperintense rim on FLAIR images. RSSIs were defined as lesions showing hyperintensity on DWI and hypointensity on apparent diffusion coefficient (ADC) maps. The number of lacunes was defined as the total number of lacunes and RSSIs. WMHs were defined as hyperintense lesions in the deep white matter or periventricular regions on FLAIR images and were graded using the Fazekas scale from 0 to 3. EPVS were identified as punctate or linear hyperintense lesions in the basal ganglia on T_2_WI and were rated using a validated 0-4 semiquantitative scale. CMBs were defined as small, round lesions with homogeneous hypointensity on SWI ^[4]^.

**Supplementary Table**

Supplementary Table 1. Main effect of position on ReHo

| Cluster index | Brain regions (AAL) | Cluster (Voxels) | Upright position | Supine position | *F* value | MNI coordinate  (X, Y, Z) |
| --- | --- | --- | --- | --- | --- | --- |
| 1 | ORBsupmed_R | 21 | 0.14 ± 0.36 | -0.12 ± 0.38 | 4.51 | (9, 66, -12) |
| 2 | ITG_L | 24 | 0.80 ± 0.51 | 0.50 ± 0.59 | 4.55 | (-48, -45, -12) |
| 3 | MFG_L | 254 | -0.01 ± 0.33 | -0.41 ± 0.25 | 5.68 | (-30, 48, 18) |
|  | IFGtriang_L | 135 |  |  |  |  |
|  | SFG_L | 85 |  |  |  |  |
|  | ORBinf_L | 37 |  |  |  |  |
| 4 | PUT_L | 20 | 0.08 ± 0.40 | -0.24 ± 0.39 | 3.87 | (-30, 6, 6) |
| 5 | SFG_R | 106 | 0.00 ± 0.31 | -0.38 ± 0.23 | 5.14 | (12, 57, 15) |
|  | SFGmed_R | 100 |  |  |  |  |
|  | MFG_R | 80 |  |  |  |  |
|  | IFGtriang_R | 55 |  |  |  |  |
|  | ACG_R | 39 |  |  |  |  |
| 6 | STG_R | 106 | 0.36 ± 0.35 | -0.04 ± 0.31 | 6.29 | (21, -45, 9) |
|  | CAL_R | 51 |  |  |  |  |
| 7 | PUT_R | 50 | 0.07 ± 0.30 | -0.29 ± 0.26 | 4.77 | (24, 6, 3) |
| 8 | SMG_R | 21 | -0.74 ± 0.34 | -0.43 ± 0.46 | -3.90 | (57, -48, 33) |
| 9 | PoCG_R | 28 | -0.52 ± 0.41 | -0.17 ± 0.49 | -4.52 | (60, -21, 36) |
| 10 | PoCG_L | 21 | -0.07 ± 0.47 | 0.23 ± 0.55 | -5.09 | (-30, -27, 72) |

X, Y, Z represent the MNI coordinates of the peak voxel within the cluster. ReHo, regional homogeneity; AAL, automated anatomical labeling atlas; MNI, Montreal Neurological Institute; ORBsupmed, medial orbital superior frontal gyrus; ITG, inferior temporal gyrus; MFG, middle frontal gyrus; IFGtriang, triangular part of the inferior frontal gyrus; SFG, superior frontal gyrus; ORBinf, orbital part of the inferior frontal gyrus; PUT, putamen; SFGmed, medial superior frontal gyrus; ACG, anterior cingulate gyrus; STG, superior temporal gyrus; CAL, calcarine cortex; SMG, supramarginal gyrus; PoCG, postcentral gyrus

Supplementary Table 2. Main effect of group and group × position interaction on ReHo at an uncorrected threshold

| Cluster index | Brain regions (AAL) | Cluster (Voxels) | Group | Upright position | Supine position | *F* value | MNI coordinate  (X, Y, Z) |
| --- | --- | --- | --- | --- | --- | --- | --- |
| The main effect of the group | | |  |  |  |  |  |
| 1 | ACG_R | 36 | NCs | -0.77±0.33 | -0.49±0.37 | 4.371 | (15, 27, 27) |
|  |  |  | CSVD | -0.22±0.38 | -0.25±0.42 |  |  |
| 2 | ACG_L | 41 | NCs | -0.71±0.43 | -0.43±0.37 | 4.245 | (-12, 30, 24) |
|  |  |  | CSVD | -0.17±0.47 | -0.09±0.5 |  |  |
| Group × position interaction | | |  |  |  |  |  |
| 1 | CAL_L | 21 | NCs | 0.56 ± 0.65 | 0.39 ± 0.55 | -3.830 | (-12, -93, 12) |
|  |  |  | CSVD | 0.18 ± 0.46 | 0.66 ± 0.57 |  |  |
| 2 | SFGmed_R | 30 | NCs | -0.44 ± 0.40 | -0.42 ± 0.36 | 4.019 | (9, 51, 24) |
|  |  |  | CSVD | 0.02 ± 0.43 | -0.53 ± 0.42 |  |  |
| 3 | ACG_R | 22 | NCs | -0.89 ± 0.39 | -0.57 ± 0.40 | 4.804 | (6, 33, 24) |
|  |  |  | CSVD | -0.40 ± 0.48 | -0.68 ± 0.45 |  |  |

X, Y, Z represent the MNI coordinates of the peak voxel within the cluster. ReHo, regional homogeneity; AAL, automated anatomical labeling atlas; MNI, Montreal Neurological Institute; CAL, calcarine; ACG, anterior cingulate; SFGmed, the medial part of the superior frontal gyrus.

Supplementary Table 3. Between-group and within-group postural differences in ReHo

| Cluster index | Brain regions (AAL) | Cluster (Voxels) | Upright position | Supine position | *t* value | MNI coordinate  (X, Y, Z) |
| --- | --- | --- | --- | --- | --- | --- |
| Upright position: CSVD > NCs | | | | | | |
| 1 | ACG_R | 35 | - | - | 5.32 | (15, 27, 27) |
| NCs: Upright > Supine | | | |  |  |  |
| 1 | IFGtriang_L | 38 | 0.21 ± 0.42 | -0.33 ± 0.32 | 5.44 | (-40, 45, 4) |
| 2 | ANG_R | 26 | -0.80 ± 0.36 | -0.30 ± 0.39 | -5.75 | (52, -57, 32) |
| CSVD: Upright > Supine | |  |  |  |  |  |
| 1 | HIP_L | 22 | -0.03 ± 0.38 | -0.32 ± 0.42 | 4.50 | (-18, -15, -15) |
| 2 | INS_R | 32 | 0.41 ± 0.50 | 0.08 ± 0.46 | 4.96 | (42, 0, 3) |
| 3 | PUT_R | 49 | 0.04 ± 0.38 | -0.4 ± 0.35 | 5.04 | (24, 3, 3) |
| 4 | IFGtriang_R | 34 | 0.13 ± 0.44 | -0.34 ± 0.35 | 5.06 | (27, 36, 3) |
| 5 | MFG_L | 220 | -0.03 ± 0.35 | -0.52 ± 0.22 | 6.34 | (-33, 48, 21) |
|  | IFGtriang_L | 64 |  |  |  |  |
|  | SFG_L | 52 |  |  |  |  |
| 6 | STG_R | 38 | -0.01 ± 0.45 | -0.37 ± 0.44 | 4.64 | (63, 18,9) |
| 7 | SFG_R | 138 | 0.02 ± 0.41 | -0.54 ± 0.32 | 4.99 | (12, 60,18) |
|  | MFG_R | 42 |  |  |  |  |
| 8 | CAL_L | 77 | -0.03 ± 0.36 | 0.47 ± 0.46 | -4.90 | (0, -93, 6) |

X, Y, Z represent the MNI coordinates of the peak voxel within the cluster. ReHo, regional homogeneity; AAL, automated anatomical labeling atlas; MNI, Montreal Neurological Institute; ReHo, regional homogeneity; NCs, normal controls; CSVD, cerebral small vessel disease; HIP, hippocampus; INS, insula; PUT, putamen; MFG, middle frontal gyrus; IFGtriang, triangular part of the inferior frontal gyrus ; SFG, superior frontal gyrus; STG, superior temporal gyrus; ANG, angular gyrus; CAL, calcarine; ACG, anterior cingulate.

Supplementary Table 4. Statistical summary of multiple linear regression models

| Dependent variables | Independent variables | Covariates | R^2^ | Adjusted  R^2^ | F value | Model  p value |
| --- | --- | --- | --- | --- | --- | --- |
| MMSE | Upright ReHo-ACG_R | age, sex, years of education, mean FD in upright or Δmean FD, and composite CSVD score | 0.459 | 0.358 | 4.527 | **0.002** |
| AVLT1+2+3 | ΔReHo-HIP_L |  | 0.425 | 0.306 | 3.570 | **0.009** |
| AVLT5 | ΔReHo-HIP_L |  | 0.328 | 0.216 | 2.929 | **0.029** |
| VFT | ΔReHo-HIP_L |  | 0.381 | 0.248 | 2.867 | **0.026** |
| ΔReHo-Left frontal regions | ΔHR |  | 0.370 | 0.259 | 3.325 | **0.011** |
| ΔReHo-PUT_R | ΔHR |  | 0.476 | 0.384 | 5.151 | **0.001** |

Δ was defined as supine minus upright. ReHo, regional homogeneity; ACG, anterior cingulate; PUT, putamen; CAL, calcarine cortex; HIP, hippocampus; MMSE, Mini-Mental State Examination; AVLT 1 + 2 + 3, immediate recall; AVLT 5, long-delay free recall; VFT, Verbal Fluency Test; HR, heart rate; CSVD, cerebral small vessel disease; FD, framewise displacement.

Supplementary Table 5. Group × ΔHR Interaction on Left Frontal ΔReHo

|  | ΔReHo-Left frontal regions | | | *R*^2^ | Adjusted  *R*^2^ | *F-*value | Model  *p-*value |
| --- | --- | --- | --- | --- | --- | --- | --- |
|  | Standardized *β* | *t*-value | *P-*value |  |  |  |  |
| Sex | -0.220 | -1.995 | 0.050 | 0.312 | 0.25 | 4.079 | **0.001** |
| Age | 0.345 | 2.797 | **0.007** |  |  |  |  |
| Years of education | 0.265 | 2.118 | **0.038** |  |  |  |  |
| Δmean FD | -0.174 | -1.570 | 0.121 |  |  |  |  |
| Group | -0.137 | -1.013 | 0.315 |  |  |  |  |
| ΔHR | 0.441 | 2.584 | **0.012** |  |  |  |  |
| Group × ΔHR | -0.527 | -2.784 | **0.007** |  |  |  |  |

Δ was defined as supine minus upright. ReHo, regional homogeneity; HR, heart rate; FD, framewise displacement.

Supplementary Table 6. Hemodynamic measures of NCs and CSVD

|  | NCs (n=30) | CSVD (n=41) | *t* value | *P* value |
| --- | --- | --- | --- | --- |
| Cerebral hemodynamics |  |  |  |  |
| ΔTotal arterial flow(mL·min^-1^) | 51.39 ± 84.79 | 37.96 ± 97.61 | 0.597 | 0.553 |
| ΔArterial mean velocity(cm·s^-1^) | 5.09 ± 5.97 | 6.4 ± 7.01 | -0.815 | 0.418 |
| ΔArterial minimum velocity(cm·s^-1^) | 6.26 ± 8.41 | 8.3 ± 10.77 | -0.848 | 0.399 |
| ΔArterial maximum velocity(cm·s^-1^) | 2.75 ± 6.41 | 3.31 ± 11.42 | -0.238 | 0.812 |
| Peripheral hemodynamics |  |  |  |  |
| ΔSBP | -7.03 ± 8.16 | -2.68 ± 10.37 | -1.879 | 0.065 |
| ΔDBP | -8.50 ± 5.47 | -6.24 ± 6.42 | -1.534 | 0.130 |
| ΔHR | -4.37 ± 6.77 | -4.34 ± 4.69 | -0.018 | 0.985 |
| ΔMAP | -8.01 ± 5.08 | -5.06 ± 6.34 | -2.076 | **0.042** |

Δ was defined as supine minus upright. NCs, normal controls; CSVD, cerebral small vessel disease; SBP, systolic blood pressure; DBP, diastolic blood pressure; HR, heart rate; MAP, mean arterial pressure.

**Reference**

1.	C. Yan, X. D. Wang, X. N. Zuo, et al. “DPABI: Data Processing & Analysis for (Resting-State) Brain Imaging. ” *Neuroinformatics* 14, no. 3(2016): 339-351.

2.	S. Ke, Y. Wang, J. Zhang, et al. “Assessing the impact of posture on brain volume in healthy subjects with a rotatable cryogen-free 1.5T superconducting MRI.” *Front Neurosci* 19, (2025):1644236.

3.	Y. Shi, M. J. Thrippleton, G. W. Blair, et al. “Small vessel disease is associated with altered cerebrovascular pulsatility but not resting cerebral blood flow.” *J Cereb Blood Flow Metab* 40, no.1 (2020):85-99.

4.	Y. Zhang, P. Chang, N. Liu, et al. “Correlation between lenticulostriate arteries and white matter microstructure changes in patients with cerebral small vessel disease.” *Front Neurosci* 17, (2023):1202538.
